# Supplementary material for: Acoustic Biosensors and Microfluidic Devices in the Decennium: Principles and Applications
Source: Micromachines (Basel). 2021 Dec 26;13(1):24. doi: 10.3390/mi13010024 (PMC8779171; doi:10.3390/mi13010024)
Supplement: Supplementary file 1 [file micromachines-13-00024-s001.zip › micromachines-1508566-SI.pdf]

Table S1. QCM biosensor applications with the corresponding bioreceptors and performance indicator

Application	Target	Bioreceptor layer	LOD	Device operation and uniqueness as reported
Proteins and bio-molecules	Total Prostate specific antigen (tPSA)/Cancer biomarker	Anti-PSA monoclonal antibody	0.29ng/mL	Matrix elimination buffer with dextran was reported to eliminate NSB of unrelated serum proteins. Detection of tPSA in 75% human serum was done[120]
	Prostate specific antigen (PSA)/Cancer biomarker	Anti-PSA monoclonal antibody	0.054μg/L	AuNP-detection antibody conjugate was used for improvement in sensitivity. Selective, simple and rapid detection of PSA was demonstrated for POC applications[97]
	Carcinoembryonic Antigen/Cancer biomarker	Anti-CEA monoclonal antibody	0.06ng/mL	Real time, label free detection of CEA was demonstrated with Graphene Oxide-Au nanoparticle coated QCM biosensor[112]
	Carcinoembryonic Antigen/Cancer biomarker	Anti-CEA monoclonal antibody	7.8pg/mL	Enzymatic biocatalytic precipitation (EBCP) was demonstrated for in situ detection sensitivity enhancement and lowering LOD [135].
	Trypsin/Pancreatic Disease biomarker	Synthetic Peptide chain (Pcc)	8.6ng/mL	Affinity of trypsin to cleave the peptide bond was exploited by using Pcc as bioreceptor for QCM. Target detection resulted in removal of bioreceptor peptide chain and hence resulted in increase in frequency of QCM[75]
	Trypsin/Pancreatic Disease biomarker	β-casein	0.48 nM	Real time, label free detection of trypsin based on cleavage of peptide bond in β-casein by target leading to rise in resonator frequency[122]
	L-Tryptophan (Trp)/Amino acid biomarker for neurological disorders	MIP with Trp template	0.73 ng/mL	Highly sensitive and selective biosensor for detection of Trp using MIP as bioreceptor was demonstrated. Detection of target from food and urine samples were tested[129]
	CD63 positive Exosomes/Extracellular vesicle neurological disease biomarker for biopsy	Mouse monoclonal biotinylated-anti-CD63 by affinity interaction	2.9×10^8 ESPs/mL	QCM-D sensor was reported for monitoring the exosome surface protein CD63 via frequency response and dissipation response[121].
	Immunoglobulin (IgG)	Anti-mouse IgG	0.20 μg/mL	Anodic Aluminium Oxide (AAO) nanostructure as top electrode of QCM was demonstrated for improvement in mass sensitivity due to enhanced immobilized antibody density[130].
	Protein-Carbohydrate Affinity Interaction	Azide carbohydrate	-	Branched chain carbohydrate chip was demonstrated in QCM for protein interaction by alkynyl branch molecule modified Au electrode to covalently bond to azide carbohydrate[134].
	Biomolecular antigen-antibody interaction	Anti-h myoglobin	-	A method to improve the surface bioreceptor immobilization capability of QCM by 4 times was reported using 3D carboxymethyl dextran (2000 kDa) [133].

	Hemoglobin in plasma	Anti-human hemoglobin polyclonal antibody	0.063mg/mL	Sensitive, label free method for detection of very low concentrations of hemoglobin in plasma was reported using QCM[123]
	Type IV collagenase/Biomarker for tumor and metastasis	Peptide (P1)	0.96ng/mL	Detection of target collagenase resulting in cleaving of peptide bioreceptor was reported[359].
	HIV-1 Glycoprotein 41 (gp41) /biomarker for HIV	Molecularly Imprinted Polymer (MIP) with peptide of gp14 as template	2ng/mL	Direct detection of HIV-1 gp14 protein with high specificity was demonstrated for testing in human urine sample[124].
	HIV-1 p24 antigen/Biomarker for HIV	Antibody specific to p24	1 ng/mL	Label free real time detection of low concentration of HIV-1 p24 was demonstrated with AuNP- detection antibody conjugate as mass amplifier [127]
	IFN-γ, TNF-α, TNF-α/Tuberculosis biomarker	Antibodies against 3 biomarkers	7.3 fg/mL (TNF-α), 6.3 fg/mL (IFN-γ), 7.8 fg/mL (IL-2)	A reusable sensor for detection was reported using AgNP- detection antibody conjugate. H_2O_2 was used to dissolve AgNP-detection antibody conjugate to regenerate the sensor[98].
	Non-structural protein-1 (NS1)/Dengue fever biomarker	Monoclonal immunoglobulin anti-NS1 (IgGNS1)	0.1μg/mL (QCM-D) 0.32 μg/mL (QCM)	Cellulose nanocrystal coated QCM electrode was reported to improve immobilization of IgGNS1 with specific detection of NS1 from serum samples using QCM and QCM-D[125].
	Plasmodium falciparum histidine rich protein-2 (PfHRP-2)/Protein biomarker for Malaria	Anti-PfHRP-2 antibody	12 ng/mL	Real time label free detection of PfHRP-2 in clinical human serum samples were demonstrated[126].
Cell adhesion, detection and interaction	Leukemia cells (CCRF-CEM cells)	Aptamer specific to leukemia cell	1160 cells/mL	Mass enhancement using amino phenylboronic acid (APBA) modified -AuNPs which could bind to the captured cell membrane protein was reported for sensitive detection of leukemia cells using QCM aptasensor [139,360].
	Breast cancer cell (MCF-7)	Chitosan functionalized Folic acid (CS-FA)	430 cells/mL	CS-FA specific to folate receptor in MCF-7 cell membrane was used with a biocompatible surface for cell adhesion and demonstrated recyclability by degradation of CS with lysozyme[140,361].
	Monitoring platelet deposition	Collagen	-	Platelet deposition on collagen coated QCM surface was monitored via frequency due to viscoelastic loading[144].
	Hemostasis kinetics	Collagen	-	Process of hemostasis involving platelet plug formation were monitored real time using human blood sample in PMMA flow cell incorporated QCM sensor[17]

	Adhesion and deforming morphology of rat mesenchymal stem cells (rMSC)	CNT/CS (Chitosan), CNT/CS/HA (hyaluronic acid), CNT/CS/FN (Fibronectin)	-	Interaction of cell with various bioreceptor layers were studied with deforming morphology of adhered cell was demonstrated due to specific interaction between membrane receptor on rMSC and bioreceptor[145]
	Cell adhesion and monitoring of cell response to anticancer drug	Rat liver epithelial cells and lung melanoma cells	-	Cell based biosensor with the cell's response to anticancer drug, α-tocopherol amidomalate (α-TAM), resulting in cell apoptosis, was monitored real time using QCM frequency and resistance response[146].
	Cell based sensor	Bovine aortic endothelial live cells (BAECs) cultured on the sensor	-	Cell based hybrid sensor combining acoustic sensor and electrochemical sensing in the same quartz substate to study cell adhesion characteristics[148].
	Cell based sensor/ Diabetic RBC adhesion study	Poly-L-lysine coated sensor with attached endothelial cells	-	Specific interaction of diabetic RBC with endothelial cells observed with QCM-D, demonstrating the viscoelastic and mechanical property difference between healthy and diabetic RBC[149,362].
	Cell based sensor/ protein-carbohydrate interaction at surface of immobilized cancer cells.	Epidermoid carcinoma cell line (A-431)/ breast adenocarcinoma cell line (MDA-MB-468)	-	Monitoring of glycosylation (attachment of carbohydrate to proteins) changes related to cancer progression and development were demonstrated by interaction with cells adhered onto polystyrene coated QCM [150].
	Cell based sensor/ protein-carbohydrate interaction at surface of immobilized cell.	polydopamine (PDA) modified electrode with captured suspended cells	-	QCM sensor with cells adhered were used to monitor glycosylation at adhered cell membrane by adding proteins like lectin[115]
	Metastatic Breast cancer cell with HER2/neu expression	Human anti HER2/neu monoclonal antibody	10 cell/mL	Polymeric nanoparticles of hydroxyethyl methacrylate (PHEMA) and ethylene glycol dimethacrylate (EDMA) coated QCM was reported to improve binding efficiency of biomolecules[116].
	Metastatic Breast cancer cell with CD44 expression	Hyaluronan (HA)	300 cells/mL (M231 cells)	QCM electrode modified with polydopamine and polyethyleneimine (PDA/PEI) films were used to bind HA which specifically detected CD44 expression on cell surface[141]
	Metastatic Breast cancer cell with Notch4 receptor expression	Notch-4 receptor antibody	12 cells/mL (MDAMB 231)	Sensitive and selective detection of metastatic cells were demonstrated by using cells with over expression of notch 4 receptor proteins using PHEMA nanoparticles coated QCM electrode [114]
DNA biosensors	Single Nucleotide Polymorphism (SNP) in p53 tumor suppressor gene	Hairpin capture probe with an external toehold domain	0.3nM	Detection of SNP in p53 gene sequence was demonstrated using SDR capture probe DNA having a toehold domain. Mass enhancement was achieved by using streptavidin coupled reporter probe that hybridized with open loop capture probe[154,363].

Single base gene mutation/ N^5, N^{10}-methylenetetrahydrofolate reductase (MTHFR) gene C677T mutation	Capture ssDNA probe specific to reporter probe	0.8nM	SDR involving a capture and reported probe DNA was utilized for simultaneous detection target mutant gene and regeneration. Detection of target initiated SDR at the toe hold domain of reporter that released the reporter probe -target complex[155].
Epidermal Growth factor receptor mutation/ drug resistance to lung cancer	ssDNA probe complementary to EGFR	1nM	Rapid, real time and label free detection of EGFR gene mutation were demonstrated by using QCM with Au electrode modified with nano porous structures[108].
Nucleic acid detection	Probe ssDNA specific to target	0.1nM	DNA templated formation of silver nanoclusters (AgNCs) using chemical reduction of Ag^+ ions localized along hybridized DNA target was reported to improve the sensitivity for detection of nucleic acid at low concentration[95].
MicroRNA-21 (miR-21)/ disease biomarker for cancer	ssDNA capture probe specific to miR-21	0.87pM	Sensitive detection of miR-21 was demonstrated by using TiO_2 NP-detection ssDNA probe conjugate with TiO_2 photocatalytic silver enhancement [96].
Nucleic acid detection/p53 gene fragment related to cancer	Probe ssDNA specific to target	0.1nM	QCM-D with self-assembled DNA nanostructure amplification using hybridization chain reaction (HCR) was demonstrated for 200 fold improvement in sensitive detection was reported for low concentrations of p53 gene fragments[151]
Hepatitis B Virus (HBV) DNA	ssDNA probe complementary to HBV DNA	-	Label free, single step detection of HBV virus was demonstrated[109]
Ehrlichia Canis (E. Canis)/bacterium in animals causing disease	ssDNA probe complementary to E. Canis genome	22 copies/μL	A simple, label free detection of E. Canis was demonstrated using PCR amplified gene sequence[156].
Vaccinia Virus	Capture probe DNA specific to virus DNA	-	Detection from amplified DNA target with optimization of denaturation technique to avoid rehybridization of amplified target virus sequence was demonstrated[161].
EBNA-1 DNA binding protein/ Epstein-Barr virus (EBV)cancer marker	Probe DNA specific to EBNA-1	50ng/mL (direct assay), 0.5ng/mL (Amplified assay)	Signal amplification with antibodies labelled with alkaline phosphatase (AP) was reported which showed 2 order of magnitude improvement in sensitivity[160]
Human Papilloma Virus type 58 (HPV-58)	Direct binding of biotinylated LAMP product to avidin coated sensor surface without any immobilized probe.	100 copies	Real time detection of product of loop mediated isothermal amplification (LAMP) of target in liquid phase QCM was demonstrated with higher sensitivity and reduced assay time[71].

	Hepatitis B Virus (HBV DNA)	DNA probe specific to target.	10^4 copies/mL	Rolling circle amplification (RCA) based QCM biosensor with faster and sensitive detection in 60mins was demonstrated[158]
	Malaria /P. falciparum and P. vivax	DNA target probe	-	Silver coated QCM as a detection method for post PCR amplification products with 30mins assay time was reported[157].
Pathogenic microorganism	E. Coli O157:H7	Anti-E-Coli antibody	-	Rapid detection of E. coli O157:H7 with assay time of 50mins was demonstrated using QCM with immobilized antibody[167].
	E. Coli O157:H7	Synthetic aptamer specific to E. Coli	1.46×10^3 CFU/mL	Rapid and label free detection of E.Coli was demonstrated by using synthetic aptamer as bioreceptor. Total detection time of 50 min was reported[166]
	Salmonella Typhimurium	Monoclonal anti-Salmonella antibody	10-20CFU/mL	Mass enhancement using AuNP-detection antibody conjugate to form a sandwich immunoassay, to detect lower concentrations of target was demonstrated[164].
	Salmonella typhimurium	Aptamer specific to target bacteria	10^3 CFU/mL	ssDNA aptamer with high affinity and specificity to target was obtained via QCM monitored SELEX and demonstrated QCM based aptasensor for detection of S. typhimurium[73]
	Salmonella typhimurium	Anti-Salmonella polyclonal antibodies	10CFU/mL	Improving the detection sensitivity by proper upright positioning of the bioreceptor antibody by using photochemical immobilization technique (PIT) was demonstrated[74].
	Maize chlorotic mottle virus (MCMV) in plants	Anti-MCMV antibody	250ng/mL	Highly sensitive and specific detection of MCMV with much faster detection in 2 hrs was reported using QCM biosensor[163].
	Influenza A Virus (H5N1, H5N3, H1N1, H1N3, and H6N1)	Molecularly Imprinted Polymer with virus template	10^5 particles/mL	Virus surface glycoprotein HA or NA determines the MIP structure cavity and by using a subset of MIP, various virus subtypes whose surface glycoproteins are a combination of HA and NA could be classified[364].
	Staphylococcus aureus/ bacteria found in nose	Lytic Bacteriophage together with monoclonal antibody against penicillin-binding protein PBP 2a	-	Distinguishing between methicillin resistant and sensitive strain of S. aureus for treatment was demonstrated using antibody against penicillin binding protein found in cell wall membrane on QCM-D biosensor[365]
	Avian Influenza Virus (H5N1)	Hydrogel with aptamer specific to AIV H5N1 surface protein hybridised with ssDNA to from polymer network.	0.0128 HAU	The detection of H5N1 virus by aptamer resulting in dissolution of crosslinker in the hydrogel leading to swelling and resonant frequency reduction was reported as detection mechanism[162]
	Avian Influenza Virus (H5N1)	Polyclonal antibodies against AI H5N1	0.0128 HAU	Enhancement in mass sensitivity to detect low concentration of target was achieved by magnetic nanobeads coated with anti-H5 detection antibodies forming a sandwich immunoassay[94]
	Avian Influenza Virus (H5N1)	ssDNA Aptamer specific to H5N1	2^{-4} HAU/50μL	Sensitive and specific detection of H5N1 virus strain in chicken tracheal swab was demonstrated using QCM electrode modified with nano porous Au film [118].

Table S2. FBAR biosensor applications with the corresponding bioreceptors and performance indicator

Application	Target	Piezo thin film/FBAR design/ frequency	Bioreceptor	LOD/ Sensitivity	Device operation and uniqueness as reported
Proteins and biomolecules	Carcinoembryonic Antigen (CEA)/Disease biomarker for cancer	10^0 inclined AlN/SMR/1.2GHz	Anti-CEA aptamer	- 2045.89 Hz cm^2 /ng	Shear mode FBAR with Q factor of 174 in liquid and 3.19% of K^2 was demonstrated for real time label free detection of CEA [173,183].
	Carcinoembryonic Antigen (CEA)/BSA Cancer biomarker	AlN/Back cavity/2.477GHz	SAM of anti-CEA antibody	- 3514 Hz cm^2 /ng	Target detection using FBAR was demonstrated by antigen-antibody interaction using SAM[198]
	Alpha-fetoprotein (AFP)/Cancer biomarker	ZnO/SMR/2.1GHz	Monoclonal anti-AFP antibody	1ng/mL -	Simple, label free , sensitive detection of cancer biomarker was demonstrated by using SMR FBAR resonator with antibody bioreceptor[194]
	Prostate Specific Antigen (PSA)/Cancer biomarker	ZnO/Back cavity/1.5GHz	Monoclonal anti-PSA antibody	- 1.5ng/cm^2	Label free detection PSA was demonstrated using dip and dry method in FBAR as gravimetric sensor with a Q factor of 800 [182].
	Prostate Specific Antigen (PSA)/Cancer biomarker	ZnO/Back cavity/2.2 GHz	PSA Antibody	- -	FBAR back cavity biosensor operating at second harmonics for sensitive, specific and label free detection of PSA was demonstrated by utilizing PSA antibody as bioreceptor [196].
	Glucose Monitoring	AlN/SMR/1.53GHz	Single wall carbon nano tubes-PDDA/Glucose oxidase composite film	0.05mM 0.84 (MHz.cm^2)/µg	Multimode sensor characterization for glucose monitoring using gravimetric and electrochemical sensing was demonstrated[15].
	Human Immunoglobulin (IgE)/ Micro allergic sensor	23^0 inclined AlN/Back cavity/1.175GHz	Anti-human IgE antibody	- 1.425 × 105 cm^2/g.	Pure shear mode FBAR biosensor with c axis tilted AlN film was reported for the detection of IgE, which is responsible of allergic reactions[190].
	Mucin 1/ Tumor marker	ZnO/SMR/1.5GHz	Streptavidin/AuNP-MUC1 aptamer chelate	20nM 4642.6 Hz/nM	Indirect method for detection of MUC-1 utilizing biotin modified aptamer- sensor immobilised streptavidin binding has been demonstrated using dip and dry method. FBAR reported a Q factor of 224 and K^2 of 2.39%[200]

	Mucin 1/ Tumor marker	AlN/SMR/575MHz	Streptavidin/AuNP-MUC1 aptamer	- 818.6 Hz/nM	Indirect method for detection of MUC-1 utilizing biotin modified aptamer- sensor immobilised streptavidin binding has been demonstrated using dip and dry method. FBAR reported a Q factor of 384 and K^2 of 3.74%[195]
	Protein ligand interaction (Biotin-Streptavidin)	ZnO/Back cavity/1.6GHz	Biotin	-	Pure shear mode FBAR using lateral filed excited shear mode has been demonstrated with Q factor of 428 in glycerol [176].
	Thrombin/Protein detection	23^0 inclined AlN/SMR/1.3GHz	Aptamer specific to thrombin	- 1800 $kHz/pg\cdot cm^2$	FBAR gravimetric biosensor for operating in liquid medium was demonstrated using c-axis inclined AlN thin film for detection of proteins[46]
	Blood Coagulation monitoring	AlN/SMR/1.9GHz	-	-	Blood coagulation monitoring was demonstrated using LFE FBAR sensor with possible use as low cost, portable miniature analytical tool[49]
	Blood Coagulation monitoring	AlN ring resonator in Contour mode/150MHZ	-	-	Blood coagulation monitoring by viscosity change due to fibrin polymerization was demonstrated using a contour mode AlN FBAR with a droplet of blood[48]
DNA biosensors	DNA synthesis	ZnO/Back cavity/ 2GHz	Probe ssDNA	-	Real time, label free monitoring of DNA synthesis was demonstrated by gravimetric method using FBAR[202].
	DNA hybridization in human serum	c-axis inclined ZnO/SMR/800MHz	Probe ssDNA	-	Real time monitoring of DNA hybridization in complex human serum was demonstrated by using a lipoamide as a blocking agent to reduce NSB from human serum[181].

Table S3. SAW biosensor applications with the corresponding bioreceptors and performance indicator

Application	Target	Mode	Substrate/Frequency	Bioreceptor	LOD/ Sensitivity	Device operation and uniqueness as reported
	Cardiac Troponin I (cTnI)/Biomarker for AMI	Love wave	36°YX $LiTaO_3$ /SiO2 waveguide layer/200MHz	Anti-cTnI capture antibody.	24.3pg/mL -	Mass sensitivity enhancement was achieved by using triple transit echo wave and AuNP conjugated detection antibody[250].
	Cardiac Troponin I (cTnI)/Biomarker for AMI	Love wave	36°YX $LiTaO_3$ /SiO_2 waveguide layer/200MHz	Anti-cTnI capture antibody	6.2pg/mL -	Sensitivity enhancement was demonstrated by sandwich immunoassay involving AuNP-detection antibody conjugate along with in situ Au staining [281].
	Myoglobin, CK-MB, and cTnI/ Cardiac Biomarker for AMI	Love wave	36°YX $LiTaO_3$ /SiO_2 waveguide layer/200MHz	Capture antibody specific to 3 biomarkers.	20 pg/mL (cTnI), 1.1 ng/mL (CK-MB) 16.0 ng/mL (myoglobin) -	Signal amplification achieved by using sandwich immunoassay using AuNP conjugated capture antibody along with Au staining for detection of multiple cardiac biomarkers [288].
Proteins and bio-molecules	Exosomes/Extracellular vesicles acting as disease biomarker alternative to liquid biopsy	Sam5 Love wave commercial sensor	Sam5 Love wave commercial sensor	Anti-CD63 antibody capture antibody	1.1×10^3 particles/mL -	Two order magnitude lower LOD was reported with sandwich immunoassay with anti EpCAM antibody-biotin conjugate and AuNP labelled streptavidin amplification[260].
	Prostate Specific Antigen (PSA)/Prostate cancer biomarker	Love wave	36°YX $LiTaO_3$ /SiO_2 waveguide layer/200MHz	Synthetic aptamer-beacon	10ng/mL -	DNA aptamer -beacon with a stem loop structure that has high affinity to target PSA was used for selective and specific detection[271].
	Prostate Specific Membrane Antigen (PSMA)/Prostate cancer biomarker	Love wave	ST-cut quartz/SiO_2 waveguide layer/160MHz	Molecularly Imprinted Polymer (MIP)	0.013ng/mL -	MIP with target PSMA template cavities was spin coated on the sensing area of the Love wave sensor to capture target PSMA [213].
	Thrombin/Protein-ligand interaction	SH-SAW	Quartz/OJ9, Japan Radio Co., Ltd.	Aptamer specific to thrombin	7.5pmol -	Achieving optimum aptamer density was demonstrated by using PEG-b-PAMA modified Au sensing area [280].

B-cell lymphoma 2 (Bcl-2)/Protein biomarker for ovarian cancer	SH-SAW	ST-900 X-Quartz/16.8MHz	Polyclonal rabbit antibody against Bcl-2.	-<br>-	Sensitive detection of Bcl-2 was demonstrated by proper orientation and density of immobilized antibody [286].
Streptavidin/ Protein-ligand interaction	R-SAW resonator	128^0YX $LiNbO_3$ /1.25GHz	Biotin-polyethylene glycol-thiol (bPEG).	104pM<br>-	Rayleigh resonator with positive and negative reflectors was demonstrated to improve the device sensitivity by trapping the acoustic energy [237].
Streptavidin/ Protein-ligand interaction	R-SAW on cantilever (CL)	GaAs/AlAs membrane cantilever/2.9 GHz SAW	Biotin at cantilever bottom surface	-<br>-	Adsorption of target at the R-SAW CL backside resulted in stress induced variation in the acoustic wave velocity that was detected by the SAW IDT on the CL top surface[238].
Dopamine (DA)/neurochemical disease biomarker	SAW resonator	Quartz/433MHz	Electrodeposited Ni-TiO2 -poly(L-lysine) composite film	0.067nM<br>-	A novel method of Electric filed assisted liquid phase oxidation was used for DA adsorption from the medium to the sensing layer[278].
Dopamine (DA)/ neurochemical disease biomarker	SH-SAW	$LiTaO_3$ /104MHz	Molecularly imprinted polymer with DA template cavities	10nM<br>0.55^0/mM	MIP with DA templates were formed with monomers and crosslinkers and the pre polymer solution was spin coated on the sensing area of SAW sensor in the delay line[292].
Dopamine (DA)/ neurochemical disease biomarker	Love wave	ST-cut quartz/SiO_2 waveguide layer	Molecularly imprinted polymer with DA template cavities	0.1pg/mL<br>-	Excellent specificity to target with low LOD was reported with novel method of MIP formation on the sensor surface with cleavable bond[293].

D-serine/ neurochemical disease biomarker	Love wave	AT-cut quartz/148MHz	D-serine dehydratase (Dsd) enzyme	950μM 0.21°/mM	Inactivated form of Dsd enzyme capable to bind target d-serine was used as bioreceptor to detect D-serine[262].
Endotoxin/ bacterial toxin causing diseases	SH-SAW	36° Y-90° X quartz/246.2 MHz	Aptamer specific to endotoxin	3.53ng/mL -	CVD SLG improved the device sensitivity and provided a biocompatible layer for bioreceptor binding and target detection[55].
Okadaic Acid (OA)/marine toxin causing shell fish poisoning in human	Love wave	AT-cut quartz/SiO_2 waveguide layer/117MHz	Anti-OA antibody	2μg -	Sandwich immunoassay using a detection was used to enhance the mass loading to improve the device sensitivity to detect small molecular mass OA[294].
Epidermal Growth factor (EGF)/Cancer biomarker	Love wave	36°YX $LiTaO_3$ /SiO_2 guiding layer/122MHz	Antibody specific to EGF	1 ng/mL 1.709kHz/(ng/mL)	Improvement in device sensitivity was demonstrated with chemisorbed layer of bioreceptor[253].
E. coli L-asparaginase/tetrameric enzyme for treatment of cancer	SH-SAW	SAM®5 Blue from SAW Instruments GmbH	Polyclonal rabbit antibody against E. coli L-ASNase	0.5pg/mm^2 -	Various antibody immobilization strategies were studied for better selectivity and linearity[277].
Uric Acid	Love wave	36°YX $LiTaO_3$ /ZnO guiding layer/52.5MHz	Uricase enzyme	5μM 766Hz/mM	Acoustoelectric effect was used in sensing where increase in resonant frequency was observed due to decrease in conductivity of medium by target bioreceptor interaction[52].
Glucose	Love wave resonator	8%Mn-ZnO/SiO_2 waveguide layer/433MHz	Glucose oxidase (GOx) and catalase enzyme covalently bonded to pH sensitive polymer P-COOH.	$4.2x10^{-4}$mM 7.184 MHz/mM	The reduction in pH of the sensing medium by enzymatic reaction between target and bioreceptor was detected by P-COOH leading to its shrinking[211].

Carcinoembryonic Antigen (CEA)/biomarker of tumor	Lamb Wave FPW	ZnO on SiO_2, Si_3N_4 membrane/10-20MHz	Anti-CEA capture antibody.	5ng/mL<br>-	FPW device with backside cavity membrane being utilized of bioreceptor immobilization with improved design of IDT to improve the IL of the FPW sensor[261].
Carcinoembryonic Antigen (CEA)/biomarker of tumor	Love wave	36°YX $LiTaO_3$ /SiO_2 guiding layer/165MHz	Anti-CEA capture antibody.	1.25ng/mL<br>0.480/(ng/mL)	CEA from exhaled breath condensate were detected by sandwich immunoassay as signal amplification method[273].
Carcinoembryonic Antigen (CEA)/biomarker of tumor	Love wave	ST-cut quartz/SiO_2 waveguide layer/120MHz	Anti-CEA antibody	0.31ng/mL<br>-	Antibody immobilization on the sensor surface was improved via self-assembled monolayer for efficient detection of target CEA [287].
Carcinoembryonic Antigen (CEA)/biomarker of tumor	Love wave	ST-cut quartz/SiO_2 waveguide layer/120MHz	Anti-CEA antibody	0.084ng/mL<br>-	Improvement in antibody immobilization was reported with sensing area coated with conducting polymer with Graphene Oxide (GO), MoS2, Au nanoparticle (NP) cluster [252].
Carcinoembryonic Antigen (CEA)/biomarker of tumor	Love wave	ST90°-X-quartz/SiO_2 waveguide layer/120MHz	Anti-CEA antibody	37pg/mL<br>-	Enhancement in the device sensitivity and amplification via sandwich immunoassay with AuNP- detection anti-CEA conjugate [258].
Intracellular pH/tumor biomarker	Love wave resonator	36°YX $LiTaO_3$ /ZnO+IrO_2 multi-guiding layer/13.91MHz	Cell culture media from H460 cancer cell was utilized to detect the pH	-<br>5.9×10^{-5} (Δf/f)/pH	Acoustoelectric effect was used in sensing where lower pH resulted in higher conductivity of IrO2 waveguide layer due to electrical corrosion [18].
C-Reactive Protein (CRP)/ biomarker for inflammation	SH-SAW	$LiNbO_3$ /59.3MHz	Anti-CRP antibody	4ng/mL<br>-	Specific detection of CRP was demonstrated with increase in the insertion loss of SH-SAW sensor due to amplitude attenuation of acoustic waves[215,282].
Antibody against Viral capsid p24/HIV infection biomarker	SH-SAW	36°Y-cut 90°X-propagation Quartz/251.5 MHz	Recombinant p24 capture ligands	165ng/mL<br>-	Smart phone connected test of HIV with a prick of blood sample with disposable SAW biosensor was demonstrated. [284].

	Antibodies against gp41 and p24 proteins/ HIV infection biomarker	SH-SAW	36°Y-cut 90°X-propagation Quartz/251.5 MHz	Recombinant capture ligands for gp24 and p24 antibodies	22.2 μg/ml(anti-p24) 25.5 μg/ml(anti-gp41) -	Device demonstrated POC capability to detect antibodies produced in human serum against HIV proteins p24 and gp41 at clinically relevant levels with overall testing time of 5min [54].
	Influenza A H1N1 virus antigen/Influenza viral biomarker	Love wave	41∘ YX $LiNbO_3$ /120MHz/SiO_2 guiding layer	Antibody specific to Hemagglutinin surface protein of H5N1.	1ng/mL -	Sensitive and specific detection of H1N1 influenza A antigen was demonstrated with impact of surface functionalization of antibodies on device performance [283].
	Chemokines/ biomarker for Respiratory Syncytial Virus (RSV)	Lamb Wave FPW	PZT on SOI/25MHz	SAM of alkyl with azide(N3) head groups that binds to chemokines protein.	- 14 Hz/nM	Sensor for POC application was demonstrated with backside cavity sensing area for FPW device[216,285].
Cell adhesion/detection and cell study	Cell Growth in 2D and 3D culture	Love wave resonator	36°YX $LiTaO_3$ /ZnO guiding layer/14.05MHz	PDMS well integrated as culture insert	- -	Microfluidic device for non-invasive quantification of human lung adenocarcinoma cell and non-cancerous cell density was demonstrated using cell-substrate adhesion[366].
	Jurkat T-lymphocyte cell detection	Love wave	36°YX $LiTaO_3$ /SiO_2 guiding layer/121.3MHz	Antibody against Jurkat cells surface protein CD3.	10^3 cells/mL -	Real time, fast detection and separation of T-cells from mixed cell medium was reported by selectively capturing the target T cell with expression of protein called CD3 [296].
	Circulating tumor cell detection/Metastatic potential of cancer determination	Leaky SAW	36°YX $LiTaO_3$ /100MHz	Aptamer specific to CTC surface protein Mucin1	32cells/mL -	Detection of metastatic potential of cancer was demonstrated by detection of CTC in human serum, by using aptamer specific to CTC surface protein Mucin1 [263].

	Cell adhesion with substrate/Tendon Stem Cell adhesion	Love wave	36°YX $LiTaO_3$ /Parylene-C guiding layer/131MHz	Collagen	-<br>-	Real time monitoring of cell adhesion to the collagen coated substrate was demonstrated using SAW sensor with variation in S21 phase and amplitude [297,300].
	Wound healing assay/cell migration	Love wave	36°YX $LiTaO_3$ /SiO_2 guiding layer/207MHz	-	-<br>-	Dynamic cell spreading and attachment was monitored real time non-invasively using a SH-SAW sensor by observing phase shift and amplitude of scattering parameters [298].
DNA biosensors	Nucleic acid detection	Sam5 Love wave commercial sensor	Sam5 Love wave commercial sensor	Capture ssDNA probe	0.8pM<br>-	Mass synergetic effect obtained by enzyme mediated DNA extension and in situ AgNP synthesis leading to signal amplification for human serum DNA detection was shown[276].
	DNA detection	SH-SAW	128^0 Y-X $LiNbO_3$ /100MHz	DNA capture probe	1.8ng/mL<br>1.2 pg/ml/Hz	A wireless transceiver was used to develop a wireless system with direct display of DNA detection results[304].
	Single Nucleotide Polymorphism (SNP)	Sam5 Love wave commercial sensor	Sam5 Love wave commercial sensor	ssDNA probe	-<br>-	Single shot assay for detection of SNP in drug metabolism enzyme gene CYP2D6*10 in clinical samples were demonstrated by using 5 channel SAM sensor system [307].
	SNP in Japanese Encephalitis Virus	Leaky SAW resonator	36°YX $LiTaO_3$ / 100MHz	Capture DNA probe	$1x10^{-12}$mol/L<br>-	Single point mutation in Japanese Encephalitis Virus is detected with enzymatic signal amplification and DNA ligation [308].
	Staphylococcus aureus (S. aureus)/ Pathogen causing respiratory infections and food poisoning	Love wave	ST-900 quartz/ SiO_2 guiding layer/282.3MHz	ssDNA probe	12.4pg/mL<br>-	Sensitive detection of S. aureus was demonstrated by using SLG-AuNP as the sensitive layer instead of Au which improved probe binding efficiency [309].
	Pseudomonas Aeruginosa/clinical pathogen causing infections in surgery/tumor	SH-SAW	36°YX black $LiTaO_3$/210MHz	ssDNA probe	0.28nmol/L<br>-	Self-assembled monolayer was utilized to immobilize probe ssDNA to improve sensitivity and lowering of LOD for target detection[214]
	Salmonella enterica serovar Typhimurium	Love wave	ST-Quartz/photoresist S1805	Direct binding of RCA	100BCE<br>-	Acoustic sensing combined with Isothermal Rolling Circle Amplification(RCA) was reported for sensitive detection [310].

			waveguide layer/155MHz	amplified target onto the sensor		
	Gene mutation detection	Leaky SAW	36°YX $LiTaO_3$ / 104MHz	Probe DNA	- -	Nearest neighbour model and real time phase shift measurement of SAW sensor were corroborated to show that sensor response showed a dependence to position of the mismatch[47].
	E. coli O157:H7	Love wave	64° YX $LiNbO_3$ /SiO_2 nanostructure waveguide layer	22-mer ss-DNA capture probe specific to E-Coli.	1.8fM 0.6439 nM/0.1 kHz	SiO_2 nanostructures were used as waveguide layer for improving bioreceptor density and provided lower LOD [311].
Pathogenic microorganism	Human Immunodeficiency Virus subtypes (HIV-1/HIV-1)	Love wave	36°YX $LiTaO_3$ /SiO_2 guiding layer/323MHz	Monoclonal antibodies specific to HIV-1 glycoprotein (gp) gp24 and HIV-2 gp39	12 TCID50s HIV-1 87TCID50s HIV-2 -	Love wave SAW sensor of 2 channels with capability to detect HIV subtypes in clinical serum samples were demonstrated [313].
	Ebola Virus	Love wave	36°YX $LiTaO_3$ /SiO_2 guiding layer	Mouse monoclonal antibody specific to Ebola surface antigen.	1.9×10^4 PFU/mL -	The sensitive and specific detection of target was reported with immobilization of antibodies specific to virus surface antigen[314].
	E-Coli	Lamb wave	4.5μm AlN deposited film on flexible PEN substrate/500 MHz	Antibody specific to E-Coli	$6.54*10^5$ CFU/mL -	Conformable, flexible lamb wave sensor was reported for detection of E-Coli with the entire sensor surface used as the sensing area [56,317].

Bacteriophage M13 (Biological Warfare agents)	Love wave	ST-cut quartz/SiO_2 waveguide layer/163MHz	Mouse monoclonal antibody anti-M13 (AM13).	-<br>-	PDMS microfluidic channels integrated sensor with reduced time for assay was demonstrated [316].
